# Preventable Diving-related Ocular Barotrauma: A Case Report

**DOI:** 10.4274/tjo.67503

**Published:** 2017-10-27

**Authors:** Serkan Ergözen

**Affiliations:** 1 ASAL Hyperbaric Oxygen Treatment Center, Undersea and Hyperbaric Medicine Clinic, Ankara, Turkey

**Keywords:** Ocular barotrauma, diving, subconjunctival haemorrhage, prevention

## Abstract

The mystical beauty of the subaquatic world is undoubtedly attractive, and many techniques and forms of equipment have been developed in the last few decades to allow us to explore the underwater world. A swimmer or diver needs swimming goggles or a diving mask to have clear vision because of the refraction problem between the eye and the water interface. Although these items are effective for clear vision, they can result in “ocular or facial barotrauma of descent” during diving. It is possible to prevent these types of barotrauma with correct techniques and precautions, thus enabling the continuation of recreational diving without recurrence. In this paper, we report a case of subconjunctival hemorrhage caused by breath-hold diving and discuss the causes of ocular barotrauma of descent and preventive measures.

## INTRODUCTION

During the last century, advances in diving techniques and equipment have made breath-hold and equipped dives more common among people who want to explore the underwater environment.

Swimmers and divers need swimming goggles or a diving mask to resolve the problem of refraction between the eye and water interface. Using these, they can see clearly when they look into water and although very useful for visual acuity under water, problems can result because of increasing pressure over the mask or goggles while diving. If the wrong equipment is used or the right technique is not implemented, the diver might experience “ocular or facial barotrauma of descent”. This case report aims to help ophthalmologists recognize subconjunctival hemorrhage due to diving activities, understand the reasons for this trauma, and present precautions to avert repeated traumas.

## CASE REPORT

A 28-year-old male presented at the outpatient clinic with the complaint of bleeding in his left eye. He reported that he had made repeated breath-hold dives to a depth of approximately 10 to 12 meters. He could equalize his ears by swallowing without any difficulties. After diving, his wife noticed rubescence in his left eye. He consulted a local ophthalmologist and was diagnosed with subconjunctival hemorrhage. No treatment was applied and he was recommended to refrain from diving again as he had a history of repeated instances of subconjunctival hemorrhage. The hemorrhage was attributed to sensitivity of the conjunctiva to something while diving. The patient did not dive again during his holiday.

**Figure 1:**
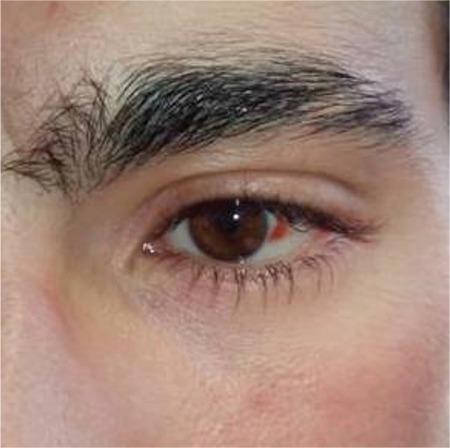
Subconjunctival hemorrhage in the patient’s left eye at presentation

After his vacation, the patient presented at our outpatient clinic to learn if this condition was related to any diving disorder. He was anxious about the recommendation to not dive again. The eye was in better condition than on the first day because subconjunctival hemorrhage heals spontaneously and quickly (Figure 1). When questioned, the patient reported no underlying disease which could explain the situation but it was learned that he had been wearing swimming goggles when diving and had a history of repeated subconjunctival hemorrhage. The patient was informed about barotrauma when diving and it was recommended that he use a diving mask instead of goggles and practice correct technique for mask equalization.

## DISCUSSION

Breath-hold diving activities (such as snorkeling and spearfishing) are becoming more popular because of the attractive environment of subaquatic world. According to Boyle’s Law, one of the physical laws of gases, there is an inverse proportion between the volume and pressure of a gas when a constant temperature is maintained.^1^ During a dive, pressure increases outside the goggles or mask but the pressure inside remains at atmospheric value, resulting in negative pressure. This negative pressure pulls the eyes and periorbital soft tissues into the goggles and can sometimes create tissue damage. When the pressure gradient is higher due to deeper dives, the likelihood of eye and periorbital soft tissue damage also increases. Tissue damage is mostly minor, such as subconjunctival hemorrhage, but in severe cases it might be more significant, such as subperiosteal hemorrhage which requires surgical intervention.^2,3,4^ Subconjunctival hemorrhage caused by diving does not require treatment and heals spontaneously, although recurrent hemorrhages resulting from recreational diving might be distressing for the patient.

This kind of ocular and periorbital soft tissue damage is well known by diving medicine physicians and can be prevented by using a diving mask instead of goggles while diving. The correct technique is to equalize the inside pressure of the mask to the outside water pressure by exhaling from the nose into the mask while descending, thereby neutralizing the vacuum effect. When ascending, the expanding volume of air will escape from the mask passively, causing no harm.^5^

Subconjunctival hemorrhage caused by diving with swimming goggles is a preventable condition. It is important that patients are questioned about goggle use if they present with subconjunctival hemorrhage after diving. When the use of goggles is determined as the cause of bleeding, the recommendation should be made to use a diving mask and equalize the pressure inside the mask to the outside water pressure by exhaling from the nose into the mask while descending.
